# ITS rDNA Gene Analysis Versus MALDI-TOF MS For Identification of *Neoscytalidium dimidiatum* Isolated from Onychomycosis and Dermatomycosis Cases in Medellin (Colombia)

**DOI:** 10.3390/microorganisms7090306

**Published:** 2019-09-01

**Authors:** Sindy V. Flórez-Muñoz, Juan C. Gómez-Velásquez, Natalia Loaiza-Díaz, Célia Soares, Carla Santos, Nelson Lima, Ana C. Mesa-Arango

**Affiliations:** 1 Grupo de Investigación Dermatológica, Instituto de Investigaciones Médicas, Facultad de Medicina, Universidad de Antioquia, Avenida Juan del Corral, 050010 Medellín, Colombia; 2 Laboratorio Clínico PROLAB, Miembro del Grupo Synlab, Calle 19 A, 050021 Medellín, Colombia; 3 CEB-Centre of Biological Engineering, Micoteca da Universidade do Minho (MUM), University of Minho, Campus de Gualtar, 4710-057 Braga, Portugal

**Keywords:** *Neoscytalidium dimidiatum*, Botryosphaeriaceae, ITS rDNA region, MALDI-TOF MS, onychomycosis, dermatomycosis

## Abstract

Within the *Neoscytalidium* genus, *N. dimidiatum*, *N. oculus*, *N. orchidacearum*, and *N. novaehollandiae* have been recognized. Although these species are frequently found in soil, *N. dimidiatum* has been identified as an etiologic agent of onychomycosis or dermatomycosis, and *N. oculus* has been identified as an etiologic agent of an ocular lesion. All these species can be cultured in vitro, but their morphological identification by macroscopic and microscopic traits is difficult and imprecise due to their similarity. In this study, 34 isolates of *Neoscytalidium* spp. from 32 onychomycosis and two dermatomycosis cases in Medellin (Colombia) were identified at the species level using sequencing of the ITS1+5.8S+ITS2 nuclear rDNA region and MALDI-TOF mass spectrometry (MS). *Neoscytalidium dimidiatum* strain MUM 17.21 was used to construct the reference spectrum in the in-house library to identify the clinical isolates by MALDI-TOF MS. Additionally, *N. dimidiatum* PPC-216 and PLAB-055 strains were used to validate the in-house constructed reference spectra. Although four groups were observed in the dendrogram obtained from the proteins of each isolate profile, MALDI-TOF MS and sequencing results are in accordance, since all isolates were identified as *N. dimidiatum*.

## 1. Introduction

The coelomycete *Neoscytalidium* (Botryosphaeriaceae) is a dematiaceous fungal genus that is commonly found in the soil of tropical and subtropical zones [1]. The taxonomy of this genus has been problematic and constantly revised. Currently, within this genus, four species are recognized: *N. novaehollandiae*, *N. orchidacearum*, *N. oculus*, and *N. dimidiatum* [2,3]. The first two species have been reported as phytopathogens, while *N. oculus* and *N. dimidiatum* have been associated to ocular or keratinized tissue (skin or nails) infections, which are indistinguishable from dermatophytosis [3,4,5]. Recently, it has been demonstrated that *N. oculus* can form biofilms and cause hemolysis [3]. In contrast, *N. dimidiatum* can also cause subcutaneous and deep infections, mainly in immunocompromised patients [6,7,8]. Furthermore, it usually shows low antifungal in vitro susceptibility with an unpredictable clinical response to treatments [9,10].

The identification of this genus has been based on the observation of macroscopic and microscopic characteristics of in vitro cultures [3]. Initially, white colonies are observed in the culture media, but with age, they become black/greenish, and the plate is fully covered with powdery aerial mycelium. Under a microscope, they are observed as being dark brown, thick-walled, with 0–2 septate arthroconidia, and variable in shape [11].

In many clinical laboratories worldwide, fungal identifications are still done by means of morphological methods, with some additional use of physiological and biochemical methods; however, they must be regarded as presumptive identifications and prone to inaccuracy. Phillips et al. [11] consider that it is inappropriate to identify fungal genera or species by only taking into account morphological characteristics, as it is prone to subjectivity. Alternatively, Matrix Assisted Laser Desorption/Ionization Mass Spectrometry with Time-of-Flight detector (MALDI-TOF MS) and gene sequencing, such as the fungal universal barcode nuclear ribosomal internal transcribed spacer (ITS) region [12], can be considered to accomplish a more objective and accurate fungal identification. The former technique has been used successfully in the identification of bacteria and yeasts [13]. However, the identification of some filamentous fungi is still a challenge, but not a restriction, depending more on the protocol and database used than on the equipment [14]. The aim of this study was to compare sequence-based identification using the complete sequences of the ITS1+5.8S+ITS2 rDNA region with MALDI-TOF MS identification of *Neoscytalidium* spp. clinical isolates.

## 2. Materials and Methods

### 2.1. Clinical Isolates

Thirty-four clinical isolates were collected between 2016–2018 from clinical samples of patients with onychomycosis (32) and dermatomycosis (2) in a mycological diagnosis laboratory (PROLAB, grupo Synlab, Medellin—Colombia) (Table 1). The observation of hyaline or pigmented septate hyphae in the direct microscopy examination of clinical samples with Chlorazol Black E (Delasco, IA, USA) was mandatory to consider a positive result and continue to the culture and subsequente analyses. The isolates were grown in Sabouraud dextrose agar with chloramphenicol (Biomerieux^®^, Paris, France) and incubated at 28 °C in the dark for up to seven days.

### 2.2. Molecular Identification

#### 2.2.1. Preparation of Mycelium for the Extraction of DNA

Isolates were grown in solid potato dextrose agar (PDA; Oxoid, Basingstoke, Hampshire, England) at 28 °C for 5 days. Then, approximately 0.2 cm^2^ of the biomass was transferred to a 1.5-mL microtube (KIMA, Piove di Sacco, Padova, Italy) containing 1 mL of yeast malt broth (YMB; yeast extract, 3 g/L; malt extract, 3 g/L; peptone 5 g/L; glucose 10 g/L). The isolates were incubated for 10 days in constant agitation at room temperature. Subsequently, they were centrifuged for 10 min at 14,000 g, the supernatant was discarded, and 1 mL of sterile distilled water was added to remove residues from the culture medium. It was centrifuged again, and the supernatant was removed, allowing the pellet to dry and later be kept at −20 °C for DNA extraction.

#### 2.2.2. DNA Extraction

DNA extraction was performed following the protocol established by Rodrigues et al. [15], with minor modifications: the biomass was transferred into Lysing matrix MP Biomedicals tubes (Santa Ana, CA, USA) containing 500 μL of lysis buffer (200 mM of Tris–HCl pH 8.5; 250 mL of NaCl; 25 mM of EDTA; 0.5% [*w*/*v*] SDS) and 300 mg of sterile glass beads of 1.25 to 1.65 mm in diameter (Sigma, St. Louis, MO, USA). Then, samples were mechanically lysed for 40 s in a FastPrep-24^TM^ 5G Instrument (MP Biomedicals Santa Ana, CA, USA); subsequently, they were incubated in a water bath for 30 min at 65 °C, and centrifuged for 10 min at 14,000× *g*. With the purpose of precipitating the polysaccharides and proteins, 500 μL of cold 3 M NaOAc pH 5.5 were added to each sample, and gently mixed by inversion before incubation at −20 °C for 10 min, and centrifugation at 14,000× *g* for 10 min. Nucleic acids were precipitated from a mix of 500 μL of the clear supernatant with 500 μL of isopropanol, which was incubated for 90 min, to finally be centrifuged at 14,000× *g* for 10 min; the precipitate was washed twice with 500 μL of cold 70% ethanol, centrifuged at 6000× *g* for 7 min, and air dried. DNA was resuspended in 50 μL of ultra-pure sterile water, quantified in a NanoDrop™ 1000 (Thermo Scientific™, Waltham, MA, USA), and stored at −20 °C.

#### 2.2.3. PCR amplification

PCR amplification of the ITS1+5.8S+ITS2 rDNA region was performed with 50 μL of a reaction mixture containing 25 μL of NZYTaq II 2x Green Master Mix (NZTtech, Lisbon, Portugal), 1 μL of primers ITS1 (5′-TCCGTAGGTGAACCTGCGG-3′) and ITS4 (5′-TCCTCCGCTTATTGATATGC-3′) [16], 1 μL of DNA, and 22 μL of sterile ultra-pure water. PCR conditions were as follows: a denaturation step at 94 °C for 3 min; 35 cycles of the annealing step: 1 min at 94 °C, 1 min at 55 °C, and 1 min at 72 °C; and a final elongation step of 5 min at 72 °C. PCR amplifications were conducted in a GeneAmp PCR System 9700 thermocycler (Applied Biosystems, Foster City, CA, USA). The products were analyzed in 1% agarose gel and purified using the NZYgelpure kit from NZYtech. Sequencing of the products was carried out in the STAB VIDA Lda (Caparica, Portugal) using the Sanger/capillary method. Sequences were processed using the FinchTV program, version 1.4.0. Poor-quality end regions were removed. ITS sequences were compared with the National Center for Biotechnology Information (NCBI) database using the Basic Local Alignment Search Tool Nucleotide (BLASTN) to detect non-specific amplicons. Only sequences with 60% coverage and at least 98% identity with *Neoscytalidium* spp. sequences were retained for further analysis.

#### 2.2.4. DNA Sequence Processing and Molecular Taxonomic Analysis

All the sequences were oriented according to the direction of the reference sequences and then globally aligned using the nested ClustalW [17] software in MEGA version 7 [18]. The best model was estimated considering the Bayesian information criterion (BIC) and the Akaike information criterion (AIC). The recovered alignments were checked manually to avoid mismatched base pairs. Reconstruction of the maximum likelihood (ML) topology with ITS1 and ITS2 sequences was performed using the Tamura-3-parameter model of nucleotide substitution, with Gamma distribution and 1000 bootstraps. Other sequences extracted from GenBank were included in the analysis, and *Scytalidium lignicola* was used as an outgroup (Table 1).

### 2.3. MALDI-TOF MS Identification

#### 2.3.1. Protein Extraction

Protein extraction was performed following the protocol of Packeu et al. [19], with minor modifications: the fungi were cultured in Sabouraud chloramphenicol agar (Biomerieux^®^, Paris, France) and incubated for 72 h at 28 °C. Mycelia were gently removed with wood sticks and suspended in a 3:1 mixture of ethanol and distilled water in a microtube of 1.5 mL (KIMA,), vortexed and centrifuged for 5 min at 13,000 rpm. The supernatants were discarded, and 50 μL of 70% formic acid was added; after being homogenized, they were left to rest for 15 min. Subsequently, 50 μL of 100% acetonitrile was added, and again, samples were allowed to rest for 15 min. Finally, they were centrifuged at 13,000 rpm for 2 min, and the supernatant was used for fungal identification.

#### 2.3.2. Constructing the Reference Mass Spectra

The reference spectra were constructed from the independent reference strain of *N. dimidiatum* MUM 17.21, obtained from the public service fungal culture collection Micoteca da Universidade do Minho (MUM, Braga, Portugal), according to the methodology proposed by Cassagne et al. [20]. Four independent cultures of strain MUM 17.21 were incubated at 28 °C for 72 h. Proteins were extracted from each culture (as described above), and 1 μL of each supernatant was transferred to a spot onto the steel target plate (Bruker Daltonics, Bremen, Germany), allowing the samples to dry completely. Then, 1 μL of α-cyano-4-hydroxycinnamic acid matrix (Bruker Daltonics, Bremen, Germany) was added. Eight replicas for each culture were used. The spectra were acquired after at least 240 shots in linear mode using the Microflex LT table mass spectrometer (Bruker Daltonics, Bremen, Germany) in the positive ion mode, with a 337-nm nitrogen laser. A bacterial standard of *Escherichia coli* extract with RNase and human myoglobin was used as control and for the internal verification of equipment calibration (BTS Bruker Daltonics, Bremen, Germany). Data were acquired automatically using the AutoXecute function of FlexControl software, version 3.3, and then exported to the MALDI Biotyper software, version 3.1 (Bruker Daltonics, Bremen, Germany), to evaluate the quality of the spectrum according to the manufacturer’s recommendations. Two isolates (PPC-216 and PLAB-055), previously identified by sequencing as *N. dimidiatum*, were used to validate the reference spectrum. *Fusarium oxysporum* ATCC 48112 and *Aspergillus fumigatus* ATCC 204305 strains were used as negative controls.

#### 2.3.3. Identification of Clinical Isolates

The protein extraction of clinical isolates was performed as described above. For each isolate, four spectra were obtained and compared to the library constructed in-house using the Maldi Biotyper 3.1 software. Identification was carried out following the manufacturer’s established scores: ≥2.000: species-level identification; 1.700–1.999: genus-level identification; ≤1.699: not reliably identified.

## 3. Results

Clinical isolates were presumptively identified as *N. dimidiatum* through classical phenotype methods and also taking into consideration that they were isolated from human patients with onychomycosis or dermatomycosis. In the beginning, all clinical isolates showed white colonies that gradually turned grey, olive green, or black. Under the microscope, mycelia that were branched, septate, hyaline, or dematiaceous with arthroconidia in chains or disarticulated, as well as occasional chlamydoconidia, were observed. In addition, molecular identification was carried out through amplification, sequencing, and phylogenetic analysis using the ITS1+5.8S+ITS2 rDNA region. The sequences of isolates included in this study were deposited in GenBank, and the accession numbers are indicated (Table 1).

**Figure 1:**
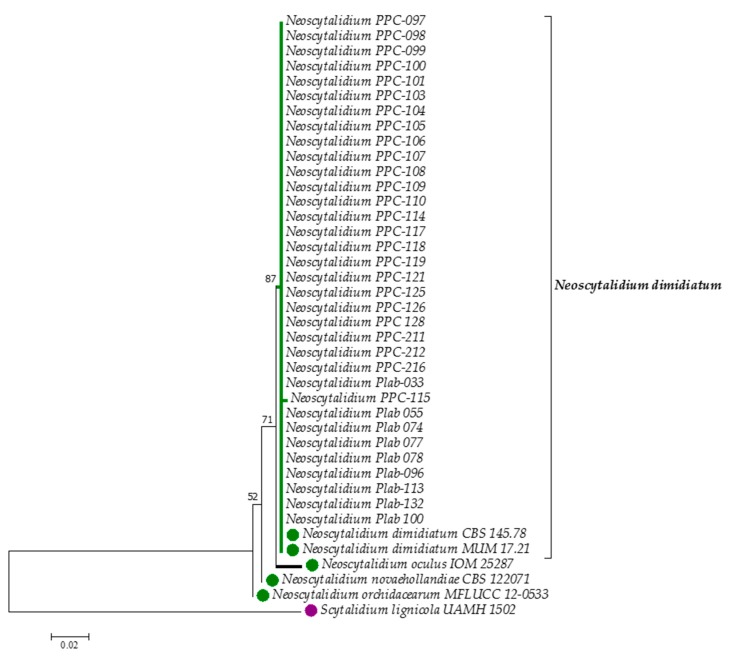
Consensus tree constructed using the maximum likelihood method with the ITS1+5.8S+ITS2 rDNA sequences of 34 clinical isolates of *N. dimidiatum*, five reference sequences of *Neoscytalidium* species (green circles), and one sequence of *S. lignicola* as the outgroup (purple circle). Percentage bootstrap values (1000 replicates) are shown next to the respective branches. Scale bar indicates nucleotide substitutions per site.

The alignment of the non-coding region ITS1+5.8SADNr+ITS2 was carried out with 40 sequences, 34 of them corresponding to clinical isolates from this study, one sequence of the ex-holotype strain of *N. dimidiatum*, the strain MUM 17.21, and three of the other species of the genus *Neoscytalidium*, and one of *S. lignicola* as outgroup (Table 1). Sequences of 407 nucleotides were used for the alignment, and they were distributed as follows: 307 conserved (75.43%); 94 variables (23.10%), of which 87 were singleton (21.38% of the total nucleotides); and six were parsimony-informative (1.47%). All positions contained gaps, and missing data were eliminated from the dataset. All the clinical isolates from this study were grouped together with the type sequence of *N. dimidiatum* in the same branch, with a bootstrap support of 87% (Figure 1). The presumptive phenotype identifications were totally confirmed by the ITS barcode.

**Figure 2:**
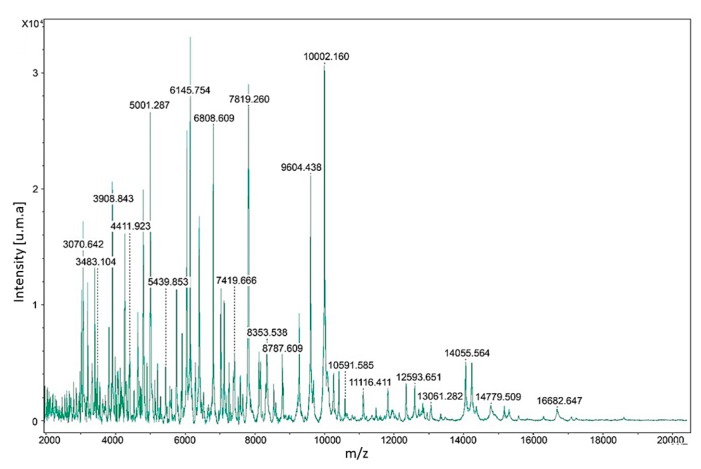
Reference spectrum of *N. dimidiatum*. On the X axis, protein mass (Dalton), and on Y, intensity units of atomic mass (u.m.a) are indicated. Numbers on peaks indicate protein mass.

For the identification of clinical isolation by MALDI-TOF MS, four reference spectra were constructed. The range of the masses detected was between 3070.643–16,682.647 Daltons (Figure 2).

**Figure 3:**
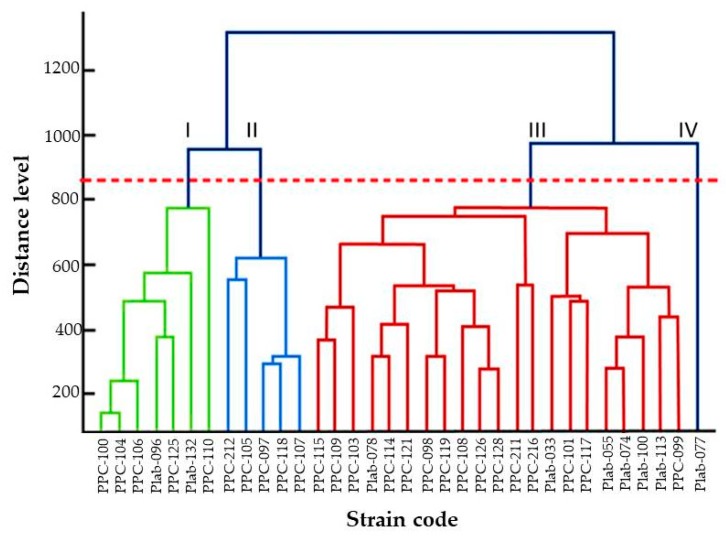
Principal component analysis (PCA) dendrogram generated by MALDI Biotyper mass spectra of 34 clinical isolates of *N. dimidiatum*. Four groups of populations are observed (I to IV) above the 850 distance level.

The 34 (100%) clinical isolates were identified through the in-house library as *N. dimidiatum* (score ≥2.00). The dendrogram created with the protein spectra, using the principal component analysis (PCA) method, shows the conformation of four groups (I to IV) above the critical distance (850), as shown in Figure 3. Groups I to III were formed by seven, five, and 21 isolates, respectively. Group IV was formed by the single isolate Plab-077 (MUM 19.71) with an origin in a nail infection.

## 4. Discussion

The constant change in the taxonomy of fungi that belong to the genus *Neoscytalidium* has generated confusion; they have been named *Dothiorella mangiferae*, *Exosporina fawcettii*, *Fusicoccum arbuti*, *F. eucalypti*, *F. dimidiatum*, *Hendersonula agathidia*, *H. cypria*, *H. toruloidea*, *Scytalidium dimidiatum*, *S. lignicola* or *Torula dimidiata* [21,22].

Traditionally, *Neoscytalidium* spp. identification has been based only on macroscopic and microscopic observations [23]. Classical phenotypic methods can give inaccurate results in the identification of the species of this genus, because they do not have characteristic structures to distinguish between them [11].

Recently, using sequencing of the ITS1+5.8S+ITS2 rDNA region, or of a fragment of the 28S large subunit ribosomal (LSU), it has been possible to recognize the species *N. novaehollandiae*, *N. orchidaceous*, *N. dimidiatum*, and *N. oculus* [3,11]. In the present study, the high level of accuracy of using the ITS1+5.8S+ITS2 rDNA region to identify *N. dimidiatum* reveals that this barcode is fit-for-propose in the clinical field. However, this barcode alone could be unable to clearly discriminate species that exhibited a certain degree of genetic variations. In this latter case, we recommended the use of a multilocus sequence analysis (MSLA). A similar situation was obtained by Pereira et al. [24] for the dermatophyte *Trichophyton rubrum*, which due to its adaptation to a highly specialized ecological niche, the skin of the human host, combined with exclusively asexual reproduction, can explain a high level of genetic uniformity within the species.

*Neoscytalidium dimidiatum* has been recognized as an etiologic agent for nail and skin infections [25,26], possibly because it produces keratinases [27]. However, with the increase of patients with immunosuppressive conditions, it has been demonstrated that *N. dimidiatum* does not only affect external tissues, but also causes eye infections or invades organs such as the lungs or the brain [6,7,8,10].

Some studies in Colombia have reported the isolation of *N. dimidiatum* or *S. dimidiatum*, as it has also been named, from skin and nail samples. In general, the identification of this fungus has been based on macroscopic and microscopic observations [5,28,29,30], with exception of the data published by Dionne et al. [7] in a case of pulmonary infection and Bueno et al. [28], who also used ITS1+5.8S+ITS2 nuclear rDNA to identify *Scytalidium hyalinum* (considered by [8] as an albino variant of *N. dimidiatum* while others [31] regard them as separate species) and *F. dimidiatum* as an etiologic agent of onychomycosis. However, the present study is the first one where *Neoscytalidium* spp. clinical isolates were identified by means of phylogenetic analysis and of MALDI-TOF MS. Phylogenetic analysis showed that all the clinical isolates were *N. dimidiatum* and grouped in one clade, separated from the other species within the same genus. In addition, it was observed that *N. oculus* is the closest related species to *N. dimidiatum*.

The construction of reference spectra was necessary because the commercial library lacks spectra for the identification of *Neoscytalidium* spp. Although the identification of filamentous fungi using MALDI-TOF MS has limitations, and even more when they are pigmented, the present study achieved both the successful construction of reference spectra and the identification of 34 clinical isolates of *N. dimidiatum*. The use of young mycelium without pigmentation (72 h of growth) might explain the correct identification by MALDI-TOF MS of all *N. dimidiatum* clinical isolates in this study. Our results are in contrast to those obtained by Alshawa et al. [32], who only achieved identification of 77.8% of the isolates. In this latter case, the low identification rate may be explained by the incubation time (three weeks), during which pigmentation was developed more intensively in combination with possible cell wall changes, such as becoming thicker and coated by hydrophobins proteins. It is known that the presence of pigments such as melanin can interfere in the identification of some fungi through MALDI-TOF MS, since the desorption/ionization process may be inhibited [33]. Also, in the identification of *Neoscytalidium* spp. by MALDI-TOF MS carried out by Alshawa et al. [32], the reference spectra of *N. dimidiatum* had seven mass peaks with an intensity >0.25 × 10^4^ u.m.a., while the ones obtained in the present study showed 34 mass peaks with an intensity >1 × 10^4^ u.m.a. In this case, both the use of young mycelia without pigments and the protein extraction method are the determining factors for adequate identification.

According to the critical distance level of 850 utilized by Chen et al. (2014) [34], the results observed in the dendrogram of Figure 3 suggest the presence of four phenotypes (Group I to IV) which shows the potential of MALDI-TOF MS to group the isolates below the species level [34]. This result contrasts that which has been observed in the consensus phylogenetic tree (Figure 2) where a single homogeneous group is observed. Although ITS1+5.8S+ITS2 rDNA is considered the universal barcoding for fungal identification, it is known that it does not have enough resolution power to detect intraspecific variability as is observed with other housekeeping gene markers, such as the elongation factor, calmodulin, or β-tubulin [12,21,35]. However, for *Neoscytalidium* spp., sequences of those genes are not available in databases for public use, as is the case of GenBank.

## 5. Conclusions

In conclusion, the results obtained with mass analysis through dendrogram construction highlights that MALDI-TOF MS can be useful in the accurate identification of strains within *N. dimidiatum* species, and is a cost-effective technique with the possibility of being used in a mycological diagnosis laboratory.

## Figures and Tables

**Table 1:** Clinical and reference fungal strains included in this study.

Strain	Collection Number	Strain Code	Origin	Country	GenBank ac. Number
*N. dimidiatum*	N/A	CBS 145.78 ^T^	onychomycosis	UK	KF531816
*N. dimidiatum*	MUM 17.21	UdeA 8023	onychomycosis	Colombia	MN371274
*N. dimidiatum*	MUM 19.44	PPC-097	onychomycosis	Colombia	MN257445
*N. dimidiatum*	MUM 19.45	PPC-098	onychomycosis	Colombia	MN257446
*N. dimidiatum*	MUM 19.46	PPC-099	onychomycosis	Colombia	MN257447
*N. dimidiatum*	MUM 19.47	PPC-100	dermatomycosis	Colombia	MN257448
*N. dimidiatum*	MUM 19.48	PPC-101	onychomycosis	Colombia	MN257449
*N. dimidiatum*	MUM 19.49	PPC-103	onychomycosis	Colombia	MN257450
*N. dimidiatum*	MUM 19.50	PPC-104	onychomycosis	Colombia	MN257451
*N. dimidiatum*	MUM 19.51	PPC-105	onychomycosis	Colombia	MN257452
*N. dimidiatum*	MUM 19.52	PPC-106	onychomycosis	Colombia	MN257453
*N. dimidiatum*	MUM 19.53	PPC-107	onychomycosis	Colombia	MN257454
*N. dimidiatum*	MUM 19.54	PPC-108	onychomycosis	Colombia	MN257455
*N. dimidiatum*	MUM 19.55	PPC-109	onychomycosis	Colombia	MN257456
*N. dimidiatum*	MUM 19.56	PPC-110	onychomycosis	Colombia	MN257457
*N. dimidiatum*	MUM 19.57	PPC-114	onychomycosis	Colombia	MN257458
*N. dimidiatum*	MUM 19.58	PPC-115	onychomycosis	Colombia	MN257459
*N. dimidiatum*	MUM 19.59	PPC-117	onychomycosis	Colombia	MN257460
*N. dimidiatum*	MUM 19.60	PPC-118	onychomycosis	Colombia	MN257461
*N. dimidiatum*	MUM 19.61	PPC-119	onychomycosis	Colombia	MN257462
*N. dimidiatum*	MUM 19.62	PPC-121	onychomycosis	Colombia	MN257463
*N. dimidiatum*	MUM 19.63	PPC-125	onychomycosis	Colombia	MN257464
*N. dimidiatum*	MUM 19.64	PPC-126	dermatomycosis	Colombia	MN257465
*N. dimidiatum*	MUM 19.65	PPC-128	onychomycosis	Colombia	MN257466
*N. dimidiatum*	MUM 19.66	PPC-211	onychomycosis	Colombia	MN257467
*N. dimidiatum*	MUM 19.67	PPC-212	onychomycosis	Colombia	MN257468
*N. dimidiatum*	MUM 19.68	PPC-216	onychomycosis	Colombia	MN257469
*N. dimidiatum*	MUM 19.69	Plab-033	onychomycosis	Colombia	MN257470
*N. dimidiatum*	MUM 19.70	Plab-055	onychomycosis	Colombia	MN257471
*N. dimidiatum*	MUM 19.71	Plab-074	onychomycosis	Colombia	MN257472
*N. dimidiatum*	MUM 19.72	Plab-077	onychomycosis	Colombia	MN257473
*N. dimidiatum*	MUM 19.73	Plab-078	onychomycosis	Colombia	MN257474
*N. dimidiatum*	MUM 19.74	Plab-096	onychomycosis	Colombia	MN257475
*N. dimidiatum*	MUM 19.75	Plab-100	onychomycosis	Colombia	MN257476
*N. dimidiatum*	MUM 19.76	Plab-113	onychomycosis	Colombia	MN257477
*N. dimidiatum*	MUM 19.77	Plab-132	onychomycosis	Colombia	MN257478
*N. orchidacearum*	N/A	MFLUCC 12-0533	environmental	Thailand	KU179865
*N. novaehollandiae*	N/A	CBS 122071	environmental	Australia	KF766207
*N. oculus*	N/A	IOM 25287	clinical	Mexico	MG764431
*S. lignicola*	N/A	UAMH 1502	environmental	Italy	NR_121314.1

^T^ = Ex-holotype strain; N/A = Not applicable.

## References

[1] Slippers B., Boissin E., Phillips A.J., Groenewald J.Z., Lombard L., Wingfield M.J., Postma A., Burgess T., Crous P.W. (2013). Phylogenetic lineages in the Botryosphaeriales: A systematic and evolutionary framework. Stud. Mycol..

[2] Huang S.K., Tangthirasunun N., Phillips A.J., Dai D.Q., Wanasinghe D.N., Wen T.C., Bahkali A.H., Hyde K.D., Kang J.C. (2016). Morphology and Phylogeny of *Neoscytalidium orchidacearum* sp. nov. (Botryosphaeriaceae). Mycobiology.

[3] Calvillo-Medina R.P., Martinez-Neria M., Mena-Portales J., Barba-Escoto L., Raymundo T., Campos-Guillen J., Jones G.H., Reyes-Grajeda J.P., Gonzalez Y.M.J.A., Bautista-de Lucio V.M. (2019). Identification and biofilm development by a new fungal keratitis aetiologic agent. Mycoses.

[4] Di Chiacchio N., Noriega L.F., Gioia Di Chiacchio N., Ocampo-Garza J. (2017). Superficial black onychomycosis due to *Neoscytalidium dimidiatum*. J. Eur. Acad. Derm. Venereol..

[5] Morales-Cardona C.A., Valbuena-Mesa M.C., Alvarado Z., Solorzano-Amador A. (2014). Non-dermatophyte mould onychomycosis: A clinical and epidemiological study at a dermatology referral centre in Bogota, Colombia. Mycoses.

[6] Bakhshizadeh M., Hashemian H.R., Najafzadeh M.J., Dolatabadi S., Zarrinfar H. (2014). First report of rhinosinusitis caused by *Neoscytalidium dimidiatum* in Iran. J. Med. Microbiol..

[7] Dionne B., Neff L., Lee S.A., Sutton D.A., Wiederhold N.P., Lindner J., Fan H., Jakeman B. (2015). Pulmonary fungal infection caused by *Neoscytalidium dimidiatum*. J. Clin. Microbiol..

[8] Garinet S., Tourret J., Barete S., Arzouk N., Meyer I., Frances C., Datry A., Mazier D., Barrou B., Fekkar A. (2015). Invasive cutaneous *Neoscytalidium* infections in renal transplant recipients: A series of five cases. Bmc. Infect. Dis..

[9] Elinav H., Izhar U., Benenson S., Admon D., Hidalgo-Grass C., Polacheck I., Korem M. (2009). Invasive *Scytalidium dimidiatum* infection in an immunocompetent adult. J. Clin. Microbiol..

[10] Yang S.J., Ng C.Y., Wu T.S., Huang P.Y., Wu Y.M., Sun P.L. (2019). Deep cutaneous *Neoscytalidium dimidiatum* infection: Successful outcome with amphotericin B therapy. Mycopathologia.

[11] Phillips A.J., Alves A., Abdollahzadeh J., Slippers B., Wingfield M.J., Groenewald J.Z., Crous P.W. (2013). The Botryosphaeriaceae: Genera and species known from culture. Stud. Mycol..

[12] Schoch C.L., Seifert K.A., Huhndorf S., Robert V., Spouge J.L., Levesque C.A., Chen W. (2012). Nuclear ribosomal internal transcribed spacer (ITS) region as a universal DNA barcode marker for fungi. Proc. Natl. Acad. Sci. USA.

[13] Dingle T.C., Butler-Wu S.M. (2013). MALDI-TOF mass spectrometry for microorganism identification. Clin. Lab. Med..

[14] SanitáLima M., Coutinho de Lucas R., Lima N., Polizeli Mde L.M., Santos C. (2019). Fungal community ecology using MALDI-TOF MS demands curated mass spectral databases. Front. Microbiol..

[15] Rodrigues P., Venâncio A., Kozakiewicz Z., Lima N. (2009). A polyphasic approach to the identification of aflatoxigenic and non-aflatoxigenic strains of *Aspergillus* section *Flavi* isolated from Portuguese almonds. Int. J. Food Microbiol..

[16] White T., Bruns T., Lee S., Taylor J., Innis M., Gelfand D., Sninsky J., White T. (1990). Amplification and direct sequencing of fungal ribosomal RNA genes for phylogenetics. PCR Protocols: A Guide to Methods and Applications.

[17] Thompson J.D., Higgins D.G., Gibson T.J. (1994). CLUSTAL W: Improving the sensitivity of progressive multiple sequence alignment through sequence weighting, position-specific gap penalties and weight matrix choice. Nucleic Acids Res..

[18] Kumar S., Stecher G., Tamura K. (2016). MEGA7: Molecular evolutionary genetics analysis version 7.0 for bigger datasets. Mol. Biol. Evol..

[19] Packeu A., Hendrickx M., Beguin H., Martiny D., Vandenberg O., Detandt M. (2013). Identification of the *Trichophyton mentagrophytes* complex species using MALDI-TOF mass spectrometry. Med. Mycol..

[20] Cassagne C., Ranque S., Normand A.C., Fourquet P., Thiebault S., Planard C., Hendrickx M., Piarroux R. (2011). Mould routine identification in the clinical laboratory by matrix-assisted laser desorption ionization time-of-flight mass spectrometry. PLoS ONE.

[21] Schoch C.L., Robbertse B., Robert V., Vu D., Cardinali G., Irinyi L., Meyer W., Nilsson R.H., Hughes K., Miller A.N. (2014). Finding needles in haystacks: Linking scientific names, reference specimens and molecular data for Fungi. Database (Oxford).

[22] Farr D.F., Elliott M., Rossman A.Y., Edmonds R.L. (2005). *Fusicoccum arbuti* sp. nov. causing cankers on pacific madrone in western North America with notes on *Fusicoccum dimidiatum*, the correct name for *Scytalidium dimidiatum* and *Nattrassia mangiferae*. Mycologia.

[23] Nkondjo Minkoumou S., Fabrizi V., Papini M. (2012). Onychomycosis in Cameroon: A clinical and epidemiological study among dermatological patients. Int. J. Derm..

[24] Pereira L., Dias N., Santos C., Lima N. (2014). The use of MALDI-TOF ICMS as an alternative tool for *Trichophyton rubrum* identification and typing. Enferm. Infecc. Microbiol. Clin..

[25] Simonnet C., Berger F., Gantier J.C. (2011). Epidemiology of superficial fungal diseases in French Guiana: A three-year retrospective analysis. Med. Mycol..

[26] Lavorato F.G., Guimaraes D.A., Premazzi M.G., Pineiro-Maceira J.M., Bernardes-Engemann A.R., Orofino-Costa R. (2017). Performance of mycology and histopathology tests for the diagnosis of toenail onychomycosis due to filamentous fungi: Dermatophyte and non-dermatophyte moulds. Mycoses.

[27] Oycka C.A., Gugnani H.C. (1998). Keratin degradation by *Scytalidium* species and *Fusarium solani*. Mycoses.

[28] Bueno J.G., Martinez C., Zapata B., Sanclemente G., Gallego M., Mesa A.C. (2010). In vitro activity of fluconazole, itraconazole, voriconazole and terbinafine against fungi causing onychomycosis. Clin. Exp. Derm..

[29] Zuluaga A., Tabares A.M., Arango M. (2001). Importancia creciente de los géneros *Fusarium* y *Scytalidium* como agentes de onicomicosis. Rev. Asoc. Col. Dermat..

[30] Zuluaga A., de Bedout C., Tabares A., Cano L., Restrepo A., Arango M. (2005). Comportamiento de los agentes etiológicos de las onicomicosis en un laboratorio de micología de referencia (Medellín 1994–2003). Med. Cutan. Iber. Lat. Am..

[31] Machouart-Dubach M., Lacroix C., Vaury C., Feuillhade de Chauvin M., Bellanné C., Derouin F., Lorenzo F. (2001). Nucleotide structure of the *Scytalidium hyalinum* and *Scytalidium dimidiatum* 18S subunit ribosomal RNA gene: Evidence for the insertion of a group IE intron in the rDNA gene of *S. dimidiatum*. FEMS Microbiol. Lett..

[32] Alshawa K., Beretti J.L., Lacroix C., Feuilhade M., Dauphin B., Quesne G., Hassouni N., Nassif X., Bougnoux M.E. (2012). Successful identification of clinical dermatophyte and *Neoscytalidium* species by matrix-assisted laser desorption ionization-time of flight mass spectrometry. J. Clin. Microbiol..

[33] Buskirk A.D., Hettick J.M., Chipinda I., Law B.F., Siegel P.D., Slaven J.E., Green B.J., Beezhold D.H. (2011). Fungal pigments inhibit the matrix-assisted laser desorption/ionization time-of-flight mass spectrometry analysis of darkly pigmented fungi. Anal. Biochem..

[34] Chen P.L., Lee T.F., Wu C.J., Teng S.H., Teng L.J., Ko W.C., Hsueh P.R. (2014). Matrix-Assisted Laser Desorption Ionization–Time of Flight Mass Spectrometry can accurately differentiate *Aeromonas dhakensis* from *A. hydrophila*, *A. caviae*, and *A. veronii*. J. Clin. Microbiol..

[35] Xu J. (2016). Fungal DNA barcoding. Genome.

